# The Effect of Er:YAG Laser Biomodification of the Implant Site Surface on Osseointegration: A Randomized Controlled Clinical Study

**DOI:** 10.3390/jfb17060287

**Published:** 2026-06-08

**Authors:** Nikolay Kanazirski, Deyan Neychev, Petya Kanazirska, Tsonka Miteva-Katrandzhieva

**Affiliations:** 1 Department of Dental, Oral and Maxillofacial Surgery, Faculty of Dental Medicine, Medical University-Plovdiv, 4000 Plovdiv, Bulgaria; deyan.neychev@mu-plovdiv.bg; 2 Department of Imaging Diagnostics, Dental Allergology and Physiotherapy, Faculty of Dental Medicine, Medical University-Plovdiv, 4000 Plovdiv, Bulgaria; petya.kanazirska@mu-plovdiv.bg; 3 Department of Social Medicine and Public Health, Faculty of Public Health, Medical University-Plovdiv, 4000 Plovdiv, Bulgaria; tsonka.miteva@mu-plovdiv.bg

**Keywords:** Er:YAG laser, dental implantology, osseointegration, primary stability, secondary stability, resonance frequency analysis, ISQ, implant site biomodification

## Abstract

**Background**: Er:YAG laser (λ = 2940 nm) biomodification of the implant osteotomy site removes the smear layer after rotary preparation and may enhance bone-implant contact. This randomized controlled clinical study evaluated implant stability dynamics following Er:YAG laser biomodification using resonance frequency analysis (RFA). **Methods:** Ninety patients were randomized 1:1 into a case group (*n* = 45; rotary osteotomy + Er:YAG biomodification; 400 mJ, 17 Hz) and a control group (*n* = 45; rotary osteotomy alone). Implant stability quotient (ISQ) was measured by RFA in vestibulo-oral (VO) and mesiodistal (MD) directions at placement, days 10, 20, 30, and month 3. **Results:** The case group showed significantly higher ISQ values at all time points in both directions (*t*-test, *p* < 0.05). Repeated measures ANOVA revealed a significant time × group interaction in the MD direction (F = 14.461, *p* < 0.001, partial η^2^ = 0.341). Primary VO ISQ: 75.04 ± 4.27 (cases) vs. 72.29 ± 3.38 (controls); primary MD ISQ: 76.49 ± 4.29 vs. 72.89 ± 2.29. The proportion achieving ISQ ≥ 70 was consistently higher in the case group. **Conclusions:** Er:YAG laser biomodification combined with rotary osteotomy yields higher, more stable ISQ values throughout early healing in mandibular D2/D3 bone, potentially supporting shorter healing intervals and early loading in selected clinical situations.

## 1. Introduction

Dental implantology has become a cornerstone of oral rehabilitation, providing predictable restoration of masticatory function and aesthetics [1,2]. The biological basis of implant success is osseointegration—the direct structural and functional connection between living bone and the implant surface under functional loading—a concept introduced by Brånemark and subsequently refined by Albrektsson and others [3,4,5]. Osseointegration depends on bone quality and quantity at the recipient site [6,7], the physicochemical properties of the implant surface [8,9,10], the surgical technique used to create the osteotomy, and the applied loading protocol [11]. Among these determinants, the method of implant site preparation has received increasing attention because it directly governs the biological environment into which the implant is introduced: it determines the thickness and composition of the debris layer on the cavity walls, the degree of thermal trauma to the surrounding bone, and the cleanliness of the interface at the moment of implant placement [12].

Placement of osseointegrable screw implants requires the creation of a precise bone bed (osteotomy). The principal challenge is heat generation, which must be kept below 47 °C to prevent thermal necrosis [3,13,14]. Three principal techniques are currently used: conventional rotary preparation, piezosurgery, and laser surgery.

### 1.1. Methods of Implant Site Preparation

The conventional rotary technique uses drills of progressively increasing diameter according to the manufacturer’s protocol. It allows precise calibration of the osteotomy and effective irrigation, but it generates an amorphous, debris-rich layer on the cavity walls—referred to throughout this manuscript as the “smear layer”—composed mainly of unmineralized collagen, proteoglycans, and bone fragments. This smear layer may impair early adhesion of blood components and delay healing [15,16], and its formation depends heavily on operator pressure and drill condition [17,18,19]. Surface biofilm formation is an additional drawback [20].

Piezosurgery, introduced clinically by Vercellotti more than two decades ago [21], allows selective cutting of mineralized tissue with reduced risk to soft tissues, less bleeding, lower noise, and a stimulating effect on peri-implant osteogenesis [22,23,24,25,26]. Its limitations include reduced precision in calibrating osteotomy length and diameter, the need for careful cooling, longer working time, and patient discomfort [22,27].

Since Maiman developed the first ruby laser in 1960, laser technology has progressively expanded its medical applications. The Er:YAG laser, emitting at 2940 nm, has become particularly relevant in dental implantology. Its wavelength is highly absorbed by water and, to a lesser extent, by hydroxyapatite, which permits precise ablation through micro-explosions in pulsed mode without carbonization [22,23,24,25,26,27,28]. Effective external water cooling produces a cleansing effect on the tissue surface during osteotomy. The reported advantages of Er:YAG-assisted implant site preparation are supported by evidence at different levels. Preclinical SEM and in vitro studies have shown that Er:YAG ablation leaves a minimal or absent smear layer (<30 µm) with precision pulsed cutting and vibration-free operation [15,29]. Animal studies have suggested that laser-prepared cavities are associated with greater bone-to-implant contact (BIC) than rotary-prepared cavities [30,31] and that Er:YAG ablation may produce a fibrin-like layer at the bone surface that adheres to the clot and is thought to facilitate cell colonization [15,32]. Clinical evidence is more limited but has reported reduced postoperative discomfort, less intra-operative bleeding, and decontamination of the cavity walls [33]. The principal drawbacks reported in the literature are imperfect dimensional accuracy of the cavity [34], potential carbonization at greater depths if cooling is insufficient [35], and longer operative time, which may necessitate general anaesthesia.

### 1.2. Rationale for the Combined Approach

To minimize the disadvantages and combine the advantages of the two techniques, our group developed a protocol that integrates conventional rotary osteotomy with subsequent biomodification of the bone surface using an Er:YAG laser at 2940 nm. In our previous studies this combined approach produced precisely shaped implant cavities with a markedly reduced or absent smear layer in an aseptic environment, as confirmed both histologically and by scanning electron microscopy (SEM) [36,37]. Briefly, split osteotomies were performed on 10 mandibles obtained from euthanized domestic pigs (Sus scrofa domestica). Group A (rotary osteotomy without laser treatment) demonstrated smear layer thicknesses of 21.81–222.13 µm; Group B (rotary osteotomy + Er:YAG laser) 6.08–43.64 µm; Group C (implant placed without laser treatment) 5.90–54.52 µm; and Group D (implant placed after Er:YAG treatment) 1.29–6.98 µm. In groups B and D, Volkmann’s and Haversian canals extended to the cavity surface, allowing direct contact between bone cells and the implant.

### 1.3. Implant Stability and Resonance Frequency Analysis

Implant stability is the principal indirect indicator of osseointegration. It is conventionally divided into primary stability (mechanical engagement of the implant in bone immediately after placement) and secondary stability (biological stability resulting from new bone formation around the implant) [38,39,40]. Among the available assessment methods, resonance frequency analysis (RFA) is the most widely used non-invasive technique [41,42]. Introduced by Meredith in 1998 and provides an objective measure of implant stability without disrupting healing [38].

In RFA, a SmartPeg is screwed onto the implant with a torque of approximately 4–6 N·cm, and the device induces vibrations at 5–15 kHz via electromagnetic pulses. The reflected resonance is converted into the Implant Stability Quotient (ISQ), defined as the resonance frequency in Hz divided by 100. Measurements are taken in two perpendicular directions—vestibulo-oral (VO) and mesiodistal (MD). ISQ < 50 indicate a mobile implant; 50–60—indicate low stability; 60–70—indicate moderate stability; ≥70—indicate high stability [43]. A typical decrease in stability is expected during the first 2–3 weeks, with the lowest values around days 28–30, when osteoclastic activity dominates [44,45].

Optimization of healing may also rely on physical, chemical, and biological modifications of the implant surface, broadly reviewed in [46,47]. Physical and chemical strategies—such as microblasting, acid etching, and anodization—have been examined in biomechanical and animal studies [48,49,50], while biological approaches based on extracellular matrix coatings have been evaluated in defect-healing models [51,52]. The critical 10–30-day post-implantation period is characterized by marginal bone resorption, declining primary stability, and as-yet absent new-bone formation, producing the well-known stability dip. Reducing this dip would allow earlier loading and shorter rehabilitation. Although the introduction of Er:YAG lasers in implantology has opened a new pathway towards this goal [53,54,55], clinical evidence remains limited.

### 1.4. Aim and Hypothesis

The aim of this randomized controlled clinical study was to evaluate the dynamics of osseointegration following Er:YAG laser biomodification of the implant site, by means of resonance frequency analysis, in comparison with conventional rotary osteotomy alone.

The working hypothesis was that the combined rotary + Er:YAG laser protocol would produce higher ISQ values and a more favourable temporal pattern of implant stability than rotary preparation alone, both at the time of placement and during early healing.

## 2. Materials and Methods

### 2.1. Study Design and Ethics

This was a single-centre, randomized, controlled, parallel-group clinical study with two arms (case and control) and two surgical sub-protocols (closed and open implantation). All patients gave their written informed consent before inclusion. The study was conducted in accordance with the Declaration of Helsinki, and the protocol was approved by the Ethics Committee of the Medical University of Plovdiv (Project identification code No. 6/5 October 2023). 

Patients meeting the eligibility criteria were assigned 1:1 to the case (Er:YAG-assisted) or control (conventional rotary) group using computer-generated block randomization (block size 4) prepared by an independent statistician. Allocation was concealed in sequentially numbered, opaque, sealed envelopes opened in the operating theatre after the patient had been prepared for surgery. Because of the nature of the intervention, the operator could not be blinded; however, the patients and the statistician performing the analysis were blinded to group assignment.

The primary outcome was the change in implant stability (ISQ) in the mesiodistal direction over time, and specifically the time × group interaction, as assessed by repeated measures ANOVA. A partial η^2^ ≥ 0.14 (large effect, Cohen 1988 [55]) was pre-specified as the threshold for clinical relevance. The secondary outcomes were: (i) ISQ in the vestibulo-oral direction; (ii) the proportion of implants achieving ISQ ≥ 70 (high stability) at each time point; and (iii) pairwise between-group differences at each measurement time point (Independent samples *t*-test).

The study was not prospectively registered. Retrospective registration was completed in the ISRCTN Registry; trial registration number: ISRCTN91819122; date of registration: 20 May 2026 (retrospective). No important changes to the trial protocol were made after the trial commenced.

No patient or public involvement was undertaken in the design, conduct, or reporting of this study.

### 2.2. Participants

#### 2.2.1. Inclusion Criteria

Patients older than 18 years, in good general health, without severe systemic conditions and without medications precluding oral surgery were eligible. All participants had previously extracted mandibular premolars or molars (extraction performed at least six months earlier, unilaterally or bilaterally), with sufficient bone volume in the premolar area and reduced bone thickness in the molar area, requiring implants up to 4.2 mm in diameter and 11.5 mm in length, without need for bone augmentation (Table 1).

#### 2.2.2. Exclusion Criteria

Patients were excluded in the presence of insufficient bone volume or density; malignancies; osteoporosis; previous radiotherapy; bisphosphonate, immunosuppressive, anticoagulant, or antiplatelet therapy; titanium hypersensitivity; pregnancy; inflammatory diseases of the oral cavity; mental illness; significant family history of implant failure; or heavy smoking (Table 1).

#### 2.2.3. Sample Size and Allocation

A total of 90 patients (47 men, 43 women) were enrolled and randomly allocated to two equally sized groups of 45 patients each. Within each group, 30 patients underwent the closed (two-stage) implantation protocol (Subtask 1), and 15 underwent the open protocol with sequential RFA measurements (Subtask 2). The mean age of the cohort was 50.99 ± 1.353 years (range 21–77 years); 57.78% had D2 and 42.22% had D3 bone density according to the Misch classification. Demographic and bone-quality characteristics did not differ significantly between the two groups (Table 2).

Recruitment and clinical work were carried out at the Department of Oral Surgery, Faculty of Dental Medicine, Medical University of Plovdiv; at the private dental practice of Dr. Kanazirski; and at the dental clinic Easy Dent, Plovdiv. The total observation period was 12 months for each patient.

### 2.3. Clinical and Paraclinical Examinations

Clinical examination included extra- and intraoral inspection, assessment of the dentition and periodontium, occlusion, mandibular range of motion, screening for parafunctions (bruxism, bruxomania), and identification of any local contraindications. All patients underwent oral cavity sanitation and professional oral hygiene (scaling and Air Flow polishing) and received instructions for personal oral hygiene before surgery.

Cone-beam computed tomography (CBCT) was performed using a Planmeca ProMax (Planmeca Oy, Helsinki, Finland) to plan implant position, dimensions, and bone density. Treatment planning was carried out in Implant Planning mode and included a panoramic curve along the alveolar ridge, axial and orthoradial reconstructions, and bone density measurement in Hounsfield units (HU) by means of segmentation in Volume Rendering mode (≤1 cm^2^ region of interest). Only patients of bone classes D2 and D3 (Misch) were included.

### 2.4. Surgical Protocols

#### 2.4.1. Implants and Operating Setup

**Figure 1:**
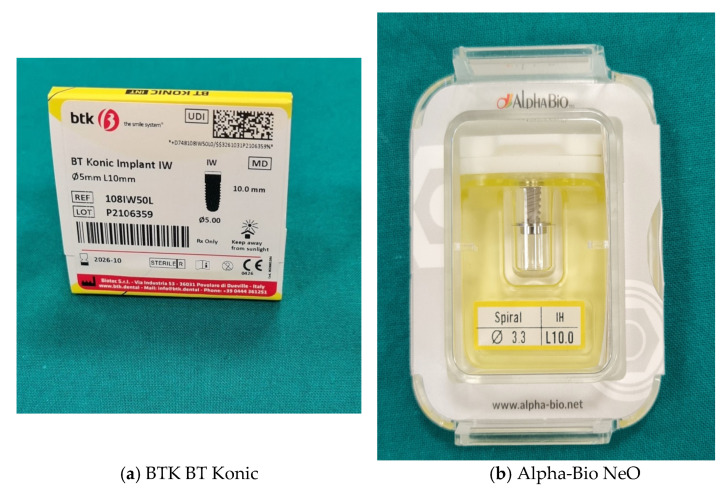
Implant systems used in the study: (**a**) BTK BT Konic (Biotec Implants, Vicenza, Italy); (**b**) Alpha-Bio NeO (Alpha-Bio Tec, Petach Tikva, Israel).

Conical osseointegrable implants from the Alpha-Bio NeO system (Alpha-Bio Tec, Petach Tikva, Israel) and the BTK BT Konic system (Biotec Implants, Vicenza, Italy) were used (Figure 1). Implants of 8, 10, and 11.5 mm length and 3.2, 3.75, 4.0, and 4.2 mm diameter were selected. To enhance comparability, implants with parameters known to produce relatively low primary stability were used. All trepanations were performed with a Bien-Air Chiropro motor (Bien-Air Dental SA, Berne, Switzerland) and a reduction implant handpiece with continuous external irrigation with sterile 0.9% NaCl. Local infiltration anaesthesia was achieved with 4% articaine + adrenaline (1:100,000 or 1:200,000).

#### 2.4.2. Case Group: Rotary Osteotomy + Er:YAG Laser Biomodification

**Figure 2:**
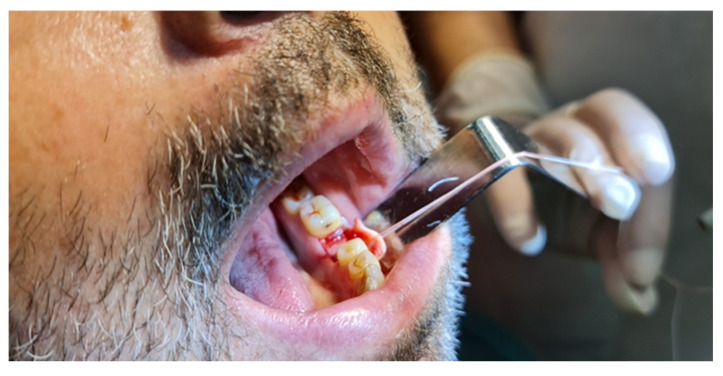
Elevated vestibular mucoperiosteal flap exposing the alveolar ridge in preparation for the osteotomy.

After intra- and extraoral disinfection, a horizontal incision was made along the alveolar ridge, slightly to the lingual side, with two relaxing incisions, where indicated. A vestibular mucoperiosteal flap was elevated using an implantology raspator (Figure 2).

Standard osteotomy was performed with the surgical kit of the corresponding system. The final drill diameter was 0.1–1.2 mm smaller than the implant diameter. The sequence consisted of marking the implant position with a round bur, pilot drilling to the planned length and depth-gauge verification, progressive widening with calibrated drills at 600–800 rpm, and shaping of the cortical entry with the corresponding profile drill.

**Figure 3:**
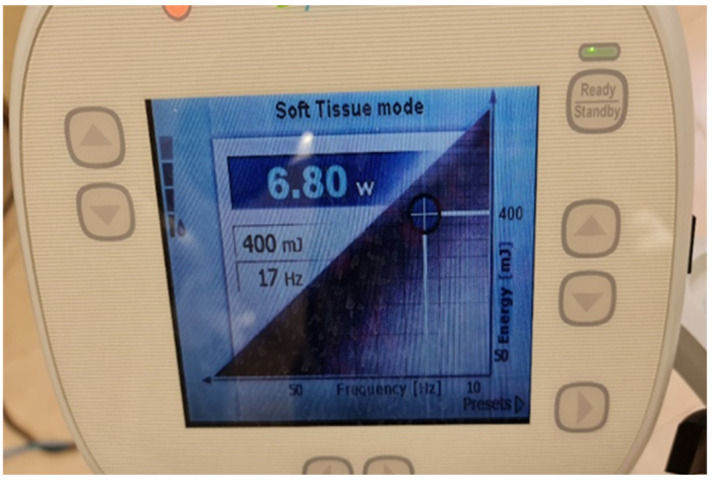
LiteTouch Er:YAG laser console (Light Instruments Ltd., Yokneam, Israel) showing the parameters used for bone biomodification: 400 mJ pulse energy, 17 Hz pulse frequency, and 6.80 W output power.

The bone surface was then biomodified with an Er:YAG laser (LiteTouch, Light Instruments Ltd., Yokneam, Israel; λ = 2940 nm). The exact laser settings used in both phases of the surgical protocol—hard-tissue (bone) biomodification and soft-tissue cutting in Subtask 2—are summarized in Table 2 to facilitate full reproducibility of the protocol. For the bone biomodification step, the program Granulation Tissue Ablation Non-contact was used. The tip emits energy at 90° to its longitudinal axis over a 180° sector; therefore, treatment was performed in two opposing quadrants (vestibular and oral) for approximately 2–3 min per quadrant, starting from the depth of the cavity and moving towards the entrance with light rotational movements (Figure 3). The same parameters were used in every patient of the case group throughout both Subtask 1 and Subtask 2.

The settings used for bone biomodification (400 mJ pulse energy, 17 Hz pulse frequency, 6.80 W output power, water spray level 6) were selected on the basis of the manufacturer’s recommended Granulation Tissue Ablation Non-contact program for the LiteTouch device, the published literature on Er:YAG ablation of mineralized tissues, and our own preclinical optimization on porcine mandibles [36,37]. Several considerations underpin these choices. First, at λ = 2940 nm the absorption coefficient of water reaches its peak (μ_a ≈ 12,000 cm^−1^), which means that, in the presence of adequate water spray, ablation occurs predominantly through micro-explosive vaporization of the interstitial water in the smear layer rather than through photothermal heating of the underlying mineralized matrix [15,28]. Second, pulse energies in the range of 200–500 mJ have been reported in in vitro and animal studies as effective for cutting bone with Er:YAG lasers without producing thermal carbonization, provided that water cooling is uninterrupted [15,30]; comparable observations have also been made in clinical and ex vivo settings [32,35]. Within this window, 400 mJ at 17 Hz produced, in our pilot SEM evaluation [37], the most consistent reduction in smear layer thickness (to <30 µm) without visible carbonization or fissuring of the cortical bone, when delivered with the AS 7631 Side-Firing tip in non-contact mode at ≈1–2 mm working distance. Third, the 17 Hz pulse frequency was preferred over higher repetition rates because, with the side-firing geometry and the manual rotational movement applied across two opposing 180° sectors, it allows sufficient inter-pulse cooling and a clinically practical total exposure of 2–3 min per quadrant. Higher pulse frequencies in pilot work tended to produce localized overheating along the cortical entry, while substantially lower frequencies prolonged the procedure without measurable benefit on smear layer reduction. Although the present trial was not designed as a parameter-optimization study, the consistency of the clinical results across all 45 case-group patients supports the appropriateness of this setting for routine D2/D3 mandibular sites; further dose–response studies remain warranted to define the optimal envelope for other bone qualities.

**Figure 4:**
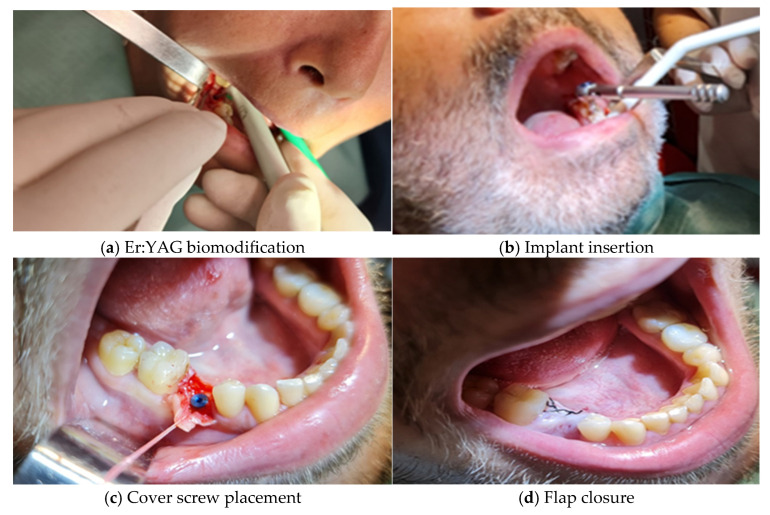
Surgical sequence in the case group: (**a**) Er:YAG laser biomodification of the prepared implant cavity using the AS 7631 (X) Side-Firing tip; (**b**) implant insertion with the calibrated torque wrench (40 N·cm); (**c**) placement of the cover screw; (**d**) flap repositioning and closure with interrupted 4/0 and 5/0 atraumatic sutures.

The implant was inserted manually with a carrier and finally torqued to 40 N·cm with a torque wrench. A SmartPeg was screwed onto the implant and primary stability was measured (see Section 2.5). After the measurement, a cover screw was placed, and the flap was repositioned without tension and sutured with interrupted 4/0 and 5/0 atraumatic sutures (Hegar holder, Figure 4).

Postoperative protocol: amoxicillin/clavulanate 875/125 mg starting one day before surgery; ibuprofen + L-arginine for analgesia. Cold compresses were recommended for the first 48–72 h. Sutures were removed between days 8 and 12. No immediate postoperative complications occurred in any of the patients in the case group.

#### 2.4.3. Control Group: Conventional Rotary Osteotomy Alone

In the control group, the surgical sequence was identical to that of the case group up to the completion of the rotary osteotomy.

Crucially, however, the bone surface of the prepared cavity was not subjected to any additional biomodification: the Er:YAG laser step was omitted. After completion of the rotary osteotomy, the implant was placed and torqued to 40 N·cm using the same protocol, and the SmartPeg was attached for primary stability measurement. Asepsis, anaesthesia, flap design, suturing, antibiotic prophylaxis, postoperative instructions, and timing of suture removal were identical to those of the case group, so that the only systematic difference between the two arms was the addition of Er:YAG biomodification in the cases.

#### 2.4.4. Subtask 2: Open (Single-Stage) Protocol

In Subtask 2 (15 cases + 15 controls), implants were placed using an open protocol that allowed repeated, non-invasive RFA measurements during early healing. Instead of a full mucoperiosteal flap, a mucosal cap of the implant diameter was excised, or a small incision along the alveolar ridge was made. In the case group, the soft-tissue cap was removed using the Er:YAG laser in a soft-tissue cutting program (settings detailed in Table 2: 200 mJ, 35 Hz, water spray level 5–6, contact mode, crystal tip 0.4 × 17 mm). In the control group, the cap was excised with a scalpel or a tissue punch. After exposure, periosteum and soft tissue were removed from the bone, and the operative sequence then matched that of Subtask 1 within the corresponding randomized arm—i.e., either rotary + Er:YAG (cases) or rotary alone (controls). After primary stability measurement, the implant was covered by an appropriately sized gingival former, allowing repeated RFA measurements at days 10, 20, and 30, and at month 3 without re-opening the surgical field.

### 2.5. Resonance Frequency Analysis

Implant stability was assessed using the Penguin RFA device (Integration Diagnostics Sweden AB, Gothenburg, Sweden). For each measurement, a sterile, autoclavable titanium SmartPeg specific to the implant system was hand-tightened with the dedicated wrench (≈4–6 N·cm). The instrument tip was held at 45° to the SmartPeg axis, 3–5 mm from the magnet, without contact. Two measurements were taken in each of the two perpendicular directions—vestibulo-oral (VO) and mesiodistal (MD)—and the mean was recorded. ISQ < 50 was considered mobile; 50–60—low; 60–70—medium; ≥70—high stability [43].

In Subtask 1 (closed protocol), primary stability was measured at implant placement, and secondary stability at the third month, after re-exposure of the implant. In Subtask 2 (open protocol), measurements were taken at placement (primary), on days 10, 20, and 30, and at month 3 (secondary).

### 2.6. Statistical Analysis

Data processing and analysis were performed using IBM SPSS Statistics, version 19 (IBM Corp., Armonk, NY, USA). A significance level of α = 0.05 was adopted for all tests, and all tests were two-tailed. Results are presented as mean ± standard deviation (SD) for continuous variables and as absolute and relative frequencies for categorical variables. The normality of the distribution of continuous variables was assessed using the Shapiro–Wilk test, and the homogeneity of variances was assessed using Levene’s test for equality of variances.

Comparisons of mean values between the two independent groups (cases vs. controls) were performed with the Independent samples *t*-test. Changes in implant stability over time, and their interaction with group assignment, were analyzed using repeated measures ANOVA, with time as a within-subject factor and group as a between-subject factor. The assumption of sphericity was assessed with Mauchly’s test, and the Greenhouse–Geisser correction was applied when violated. Effect sizes are reported as partial eta squared (partial η^2^) for ANOVA models and as Cohen’s d for pairwise comparisons. Conventional benchmarks were used: η^2^ = 0.01 small, 0.06 medium, 0.14 large [56]; d = 0.20 small, 0.50 medium, 0.80 large [57].

Reporting post hoc power values after statistically significant results have been obtained adds limited inferential value, because the probability of rejecting a false null hypothesis is already implicit in the observed *p*-values; the analysis is therefore presented solely as a standardised metric of effect magnitude to facilitate comparison with future studies, and not as a justification of the sample size.

**Figure 5:**
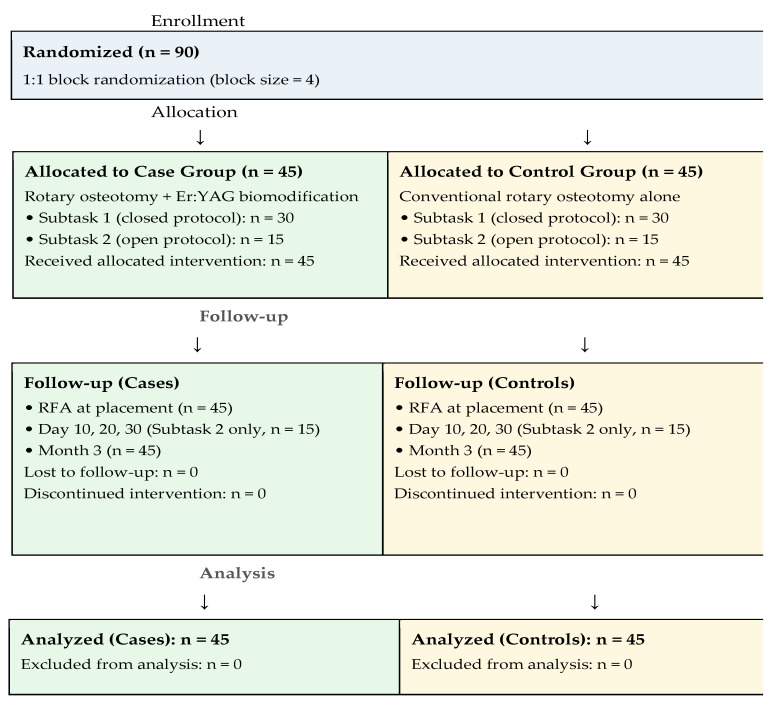
CONSORT 2025 flow diagram of the randomized controlled trial.

The study was conducted and reported in accordance with the CONSORT 2025 guidelines for randomized controlled trials [58]. The flow of participants through the trial is summarized in Figure 5.

All 90 randomized patients were analysed in their allocated group (intention-to-treat principle); no participants were excluded from the analysis. No missing data were encountered; therefore, no imputation methods were required.

It is acknowledged that a linear mixed-effects model (LMM) would offer greater flexibility in handling the repeated-measures structure of the data, particularly in studies with missing observations or complex covariance structures. In the present cohort, however, there were no missing data (lost to follow-up = 0 in both arms), removing the primary practical advantage of LMM over RM-ANOVA for this balanced, complete dataset. The Greenhouse–Geisser correction was applied when the sphericity assumption was violated (Mauchly’s test), providing a robust parametric approach appropriate for the current data structure. Future studies with larger and potentially unbalanced datasets are encouraged to employ LMM as the primary analytical framework.

## 3. Results

### 3.1. Patient Characteristics

A total of 90 patients with previously extracted mandibular premolars or molars (extraction performed at least six months earlier) completed the study and were included in the analysis. Demographic, age, sex, and bone-density characteristics are summarized in Table 3.

Both implant systems (Alpha-Bio NeO and BTK BT Konic) were used in both the case group and the control group. The Alpha-Bio NeO system was used in the majority of patients in both arms; the BTK BT Konic system comprised the remaining cases, distributed proportionally between groups. No statistically significant difference in primary ISQ values was observed between patients receiving Alpha-Bio NeO versus BTK BT Konic implants within either group (Independent samples *t*-test, *p* > 0.05 for both directions), indicating that implant type did not act as a significant confounding factor in the analysis.

### 3.2. Implant Stability—Vestibulo-Oral Direction

Implant stability values measured by RFA in the vestibulo-oral (VO) direction are presented in Table 4 for the cases and the controls at each time point.

At every time point, mean ISQ values in the VO direction were higher in the case group than in the control group, and the differences were statistically significant (Independent samples *t*-test, *p* < 0.05). The proportion of implants reaching ISQ ≥ 70 was consistently higher in the case group than in the controls (Table 3).

The between-group mean differences (MD) with 95% confidence intervals were as follows. For primary stability: VO direction, MD = 2.75 ISQ units (95% CI: 1.14, 4.36); MD direction, MD = 3.60 ISQ units (95% CI: 2.16, 5.04). For secondary stability: VO direction, MD = 3.38 ISQ units (95% CI: 1.89, 4.87); MD direction, MD = 4.13 ISQ units (95% CI: 2.73, 5.53). The most pronounced between-group difference was observed at day 20 (the nadir of the stability dip): VO direction, MD = 10.06 ISQ units (95% CI: 6.67, 13.45); MD direction, MD = 10.47 ISQ units (95% CI: 7.11, 13.83). Cohen’s d for primary stability in the VO direction was 0.71 (95% CI: 0.29, 1.14), indicating a medium-to-large standardised effect. The partial η^2^ for the time × group interaction in the MD direction was 0.341 (95% CI: 0.143, 0.539), representing a very large effect by conventional benchmarks [56,57].

### 3.3. Implant Stability—Mesiodistal Direction

The corresponding values in the mesiodistal (MD) direction are presented in Table 5.

Repeated measures ANOVA showed a statistically significant main effect of time (*p* < 0.001) for the MD direction, indicating significant change in implant stability over the follow-up period. A statistically significant time × group interaction was also observed (F = 14.461, *p* < 0.001, partial η^2^ = 0.341), confirming that the temporal pattern of stability differed between cases and controls. The Independent samples *t*-test demonstrated significantly higher ISQ values in the case group at each time point (*p* < 0.05). Descriptive analysis showed that cases maintained higher and more stable values throughout the observation period, whereas the control group exhibited a more pronounced decline between days 10 and 30.

### 3.4. Distribution of Stability Categories (ISQ ≥ 70)

The proportion of implants achieving high stability (ISQ ≥ 70) is presented in Table 6 for both directions and both groups.

In particular, between days 20 and 30—the period of the well-known stability dip—the case group preserved a markedly higher proportion of implants in the high-stability category (≥70) compared with controls in both directions. This is the period in which the most pronounced clinical effect of the Er:YAG biomodification was observed.

### 3.5. Graphical Summary of Stability Dynamics

**Figure 6:**
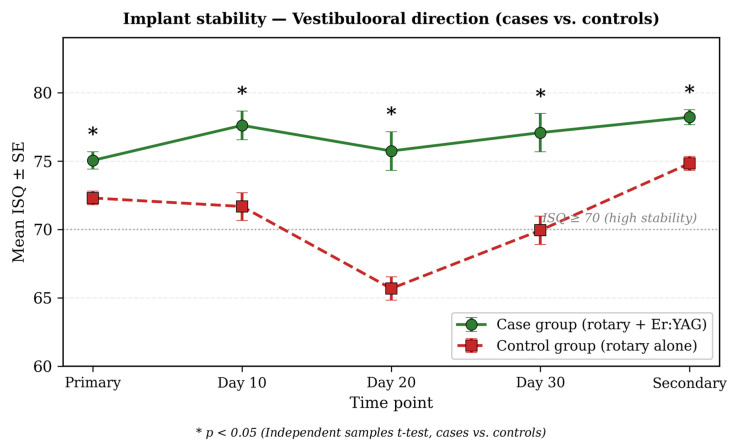
Mean implant stability quotient (ISQ ± SE) in the vestibulo-oral direction at primary, day 10, day 20, day 30, and secondary measurements—cases (rotary + Er:YAG) vs. controls (rotary alone). The case group maintains higher and more stable values throughout the observation period, while the control group shows a pronounced dip between days 10 and 30. * *p* < 0.05 (Independent samples *t*-test, cases vs. controls).

**Figure 7:**
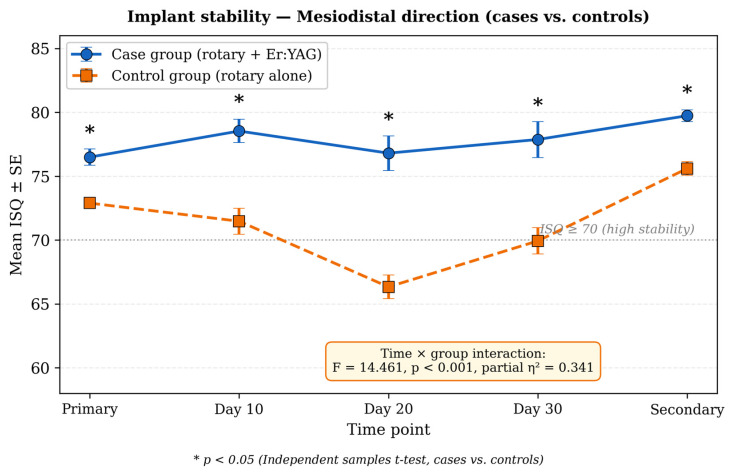
Mean implant stability quotient (ISQ ± SE) in the mesiodistal direction at primary, day 10, day 20, day 30, and secondary measurements—cases (rotary + Er:YAG) vs. controls (rotary alone). The control group shows a marked stability dip between days 20 and 30 that is largely abolished in the cases (time × group interaction: F = 14.461, *p* < 0.001, partial η^2^ = 0.341). * *p* < 0.05 (Independent samples *t*-test, cases vs. controls).

The temporal patterns of mean ISQ values for cases and controls in the vestibulo-oral and mesiodistal directions are illustrated in Figure 6 and Figure 7.

In summary, the case group consistently exhibited higher implant stability than the control group, with significantly different temporal dynamics in the MD direction; the same pattern was observed for the VO direction.

## 4. Discussion

Our previous histological and SEM studies demonstrated that the Er:YAG protocol developed by our group effectively removes the smear layer from the prepared implant bed, providing both cellular and morphological evidence to support clinical translation [36,37]. In the present RCT, the same protocol was assessed against conventional rotary preparation alone using resonance frequency analysis (RFA), showing that Er:YAG laser biomodification yields higher and more stable ISQ values throughout early healing.

Primary stability is, by definition, predominantly a mechanical phenomenon governed by the friction force between the implant and the surrounding bone, F = k·N, where k is the coefficient of friction and N the normal pressure between the surfaces [59,60,61]. Factors that increase k or N—such as implant macrogeometry, bone density, and a precise undersized osteotomy—are therefore expected to enhance primary stability [60]. By removing the smear layer, which is essentially soft, unmineralized material, the Er:YAG biomodification eliminates a yielding interface between the rigid implant surface and the underlying mineralized bone. This reduces the potential for micro-rotational movements at the time of implant placement, which are critical for implant survival according to several studies [62,63,64]. The higher primary stability in the case group (Table 4 and Table 5) is consistent with this mechanism.

Secondary stability emerges from bone regeneration and remodelling around the implant, and depends critically on initial primary stability [63]. It is predominantly a biological phenomenon—new bone synthesis around the implant. Kasemo and Lausmaa proposed that smear-layer removal and direct osteoblast contact with the implant surface may accelerate new bone synthesis through osteoconduction [54].

Secondary stability emerges from the regeneration and remodelling of bone tissue around the implant after placement, and depends critically on the initial primary stability [53]. Unlike primary stability, secondary stability is predominantly a biological phenomenon—the synthesis of new bone around the implant in competition with the surface microbial biofilm. From a surface-science perspective, Kasemo and Lausmaa proposed that the reduced compressive force on the cavity walls after removal of the smear layer, together with direct contact of osteoblasts with the implant surface, may accelerate new bone synthesis through osteoconduction [54]. Experimental work and animal studies suggest that laser-modified bone surface promotes greater BIC values [65], though clinical translation relies on a smaller, less homogeneous evidence base [66,67]. Our SEM data showed Er:YAG biomodification reduces smear-layer thickness from >100 μm to <30 μm, exposing Volkmann’s and Haversian canals [36,37]; animal studies report faster early bone healing in laser-prepared cavities [65], though effect sizes vary. Kesler et al. [33] support this trend; Stubinger et al. [35] found comparable osseointegration in a sheep model, and the present trial extends the evidence to a randomized clinical setting in human mandibular bone.

Of particular clinical interest is the period between the tenth and thirtieth day after implantation, when graphical analysis typically shows a drop in total stability—the so-called stability dip. In our cohort, the controls clearly displayed the classical pattern: day 20 mean ISQ values of 65.67 ± 0.860 (VO) and 66.33 ± 0.929 (MD), with only 13.30% of control implants reaching ISQ ≥ 70 in the MD direction. In contrast, in the case group the dip was largely attenuated: day 20 means were 75.73 ± 1.416 (VO) and 76.80 ± 1.353 (MD), with 86.70% of case implants in the high-stability category at the same time point. The repeated measures ANOVA in the MD direction confirmed that this difference was not a chance fluctuation but a systematically distinct temporal pattern between the two groups (time × group interaction F = 14.461, *p* < 0.001, partial η^2^ = 0.341, corresponding to Cohen’s f = 0.72—a very large effect by conventional benchmarks). A consistent trend was observed in the VO direction. Notably, case-group ISQ values at day 20 (75.73/76.80) approached levels reported for fully osseointegrated implants at three months, suggesting that the laser-assisted preparation substantially mitigates the third-week stability disadvantage

From a translational perspective, the goal in implantology is a stability curve that runs parallel to the timeline, i.e., without a marked dip; this objective has been pursued through a number of distinct strategies, including the optimization of laser wavelengths for implant site preparation [68,69,70], modified loading protocols [71,72,73,74], and refinements of drilling sequences and implant macrogeometry [75,76,77,78,79,80,81]. Several manufacturers attempt to approach this goal through hydrophilic implant surfaces (e.g., SLA active) [75,76]. Our results suggest that surface biomodification of the bone bed, rather than—or in addition to—the implant surface, may also contribute to this objective. Maintaining ISQ values close to or above the secondary stability level by day 30 supports the clinical possibility of early or even immediate definitive loading of implants in carefully selected cases.

In addition to its mechanical and bone-biological effects, Er:YAG treatment also produces decontamination of the osteotomy surface, eliminating the surface biofilm encountered during oral cavity surgery [76]. This contributes to reduced inflammatory responses and lower postoperative pain, which we observed clinically in the cases [77]. The principal disadvantages of Er:YAG—assisted preparation are potential carbonization at greater depths if water cooling is suboptimal, and a longer operative time, which may be less well tolerated by some patients [68]. 

### 4.1. Strengths and Limitations

The principal strength of the present study is its randomized, parallel-group, controlled design, which directly addresses the main shortcoming of the published literature on Er:YAG-assisted implant site preparation—the relative scarcity of head-to-head comparisons against conventional rotary osteotomy alone. Block randomization with allocation concealment, the inclusion of two surgical sub-protocols (closed and open), and the standardization of operator, instrumentation, antibiotic prophylaxis, and follow-up schedule jointly minimize selection and performance bias and allow both endpoint and longitudinal analyses to be performed on the same cohort. The relatively large effect size obtained for the time × group interaction in the mesiodistal direction (partial η^2^ = 0.341) further indicates that the observed differences are clinically as well as statistically meaningful.

Several limitations should nevertheless be acknowledged. First, and most importantly, performance blinding of the operator was not feasible: the visual and auditory characteristics of the Er:YAG laser step—the audible pulse, water-spray plume, and the dedicated handpiece—inevitably reveal group assignment to the surgeon during the procedure. This is inherent to any laser-vs-non-laser clinical comparison. Mitigating measures included patient blinding, a single standardised operator for both arms following an identical written protocol, objective digital RFA readout, and statistician blinding until database lock. Residual performance bias cannot be excluded, and multi-operator replication is warranted.

Second, the study was conducted at a single centre, with three closely related clinical sites supervised by the same surgical team. Although this enhances internal consistency, it limits the external generalizability of the results to other geographical, demographic, and educational settings. Third, the primary follow-up endpoint was set at three months, which corresponds to the conventional time for measuring secondary stability and exposing two-stage implants but is shorter than the period typically required to assess marginal bone level changes, peri-implant soft-tissue stability, and prosthetic survival. Long-term, multi-centre follow-up of patients restored with definitive prostheses is therefore needed to confirm whether the observed early advantage of the laser-assisted protocol translates into a sustained clinical benefit.

Fourth, in the present clinical phase no histological or histomorphometric confirmation of the bone–implant interface was obtained, since obtaining trephine biopsies in successfully osseointegrated dental implants raises ethical and clinical concerns. The mechanistic hypothesis—namely that Er:YAG biomodification reduces the smear layer and improves bone–implant contact—has, however, been documented in our previous histological and SEM studies on porcine mandibles using the same laser parameters [36,37,78,79,80].

Fifth—and most importantly from a translational perspective—resonance frequency analysis was the sole modality used for stability assessment in the present study. RFA is widely accepted as the standard non-invasive surrogate for monitoring implant stability over time [40,41], but it must be emphasized that ISQ values are an indirect mechanical proxy for the underlying biological process of osseointegration rather than a direct measurement of bone–implant contact (BIC) or new bone formation [81,82]. Documented limitations apply equally to both arms: ISQ values are influenced by implant macrogeometry, marginal bone level, SmartPeg torque, and probe angle, and the conventional ISQ ≥ 70 threshold is observational rather than histologically validated. RFA cannot distinguish primary mechanical engagement from secondary biological anchorage when both yield similar readings. The convergent findings across the two perpendicular directions (VO and MD), the consistent pattern at all five time points, the large effect size of the time × group interaction (partial η^2^ = 0.341), and the agreement between the present clinical findings and our previous histological/SEM evidence [36,37] together suggest that the observed differences are likely to reflect a genuine biological phenomenon rather than a measurement artefact, although direct biological confirmation remains outside the scope of RFA. Future studies should therefore ideally combine RFA with complementary modalities—in particular, longitudinal CBCT-based assessment of marginal bone level changes around the implant [83,84], micro-CT or histomorphometric BIC quantification in animal models, and biomarker analyses of peri-implant crevicular fluid—to triangulate the mechanistic interpretation of the ISQ data. It must be emphasised that higher ISQ values reflect greater mechanical anchorage of the implant—they do not constitute direct evidence of accelerated histological osseointegration, enhanced marginal bone formation, or clinical survival, none of which were endpoints of the present study [81,84,85].

Sixth, the sample size was determined on pragmatic clinical grounds rather than through a formal a priori power calculation. The post hoc power analysis reported in Section 2.6 indicated that the study was adequately powered for both the primary endpoint (interaction effect, achieved power > 0.99) and the principal pairwise comparisons (achieved power > 0.90), supporting the reliability of the present inferences. Even so, a formally powered follow-up study would strengthen any subsequent claims about subgroup effects or smaller incremental gains.

Finally, the study was not prospectively registered in a public clinical-trials registry. Although registration is not yet uniformly required for surgical RCTs, retrospective registration is encouraged by both ICMJE and MDPI editorial policy and would further enhance methodological transparency for future replications.

### 4.2. Clinical Implications and Translational Outlook

These findings have direct implications for everyday implantology practice. Conventional protocols for delayed prosthetic loading rest on the assumption that the early-healing stability dip described above is largely unavoidable and must therefore be “waited out” for three to six months before functional load is applied. Our data challenge this assumption in a defined clinical context: in mandibular sites of D2/D3 bone density, where biomechanically marginal cases are most often encountered, the addition of an Er:YAG biomodification step is sufficient to keep ISQ values in the high-stability range throughout the entire 30-day critical window. The clinical question therefore shifts from whether the dip can be avoided to whether the conditions under which it has been engineered out are reproducible enough to justify shorter healing intervals—and, by extension, modified loading protocols [69,71,86].

Three concrete clinical scenarios benefit most directly from this finding. The first is the patient with limited tolerance of long edentulous intervals: in our cohort the case group reached ISQ values compatible with definitive loading at day 30, opening a window for early functional or even immediate definitive loading in selected D2/D3 mandibular sites. The second is the patient at elevated risk of micromotion: bruxers, patients with cantilevered restorations, and patients in whom an undersized osteotomy is required for adequate primary engagement all benefit from an absent or attenuated stability dip, which provides a measurable biomechanical safety margin precisely when the implant is most vulnerable. The third is the immediate post-extraction site or any cavity with a low-grade microbial burden: the ablative effect of the Er:YAG step decontaminates the cavity walls in addition to removing the smear layer, addressing a problem that conventional rotary preparation does not. Each of these scenarios is distinct enough—in patient selection, surgical timing, and prosthetic design—to warrant its own dedicated prospective trial.

From a public-health and access perspective, the practical attraction of this protocol is that it does not depend on novel biomaterials, custom implants, or expensive consumables [87,88]. The Er:YAG laser unit used here is already in routine clinical use in many dental practices for soft-tissue applications, and the additional treatment time per implant is on the order of a few minutes. Any subsequent translation of the observed early-stability advantage into shorter healing intervals therefore has the potential to expand access to fixed prosthetic rehabilitation—in particular for elderly patients and patients with reduced tolerance of provisional removable prostheses—without significant capital investment by the treating practice.

Several confirmatory steps are required before this protocol can be recommended as a generalizable refinement of the standard implant placement workflow. First, multicentre validation involving multiple operators is needed to assess between-operator reproducibility of the laser application and to test the protocol’s external generalizability outside our centre. Second, long-term prospective studies should track marginal bone level changes around the implant by means of standardized longitudinal radiographic assessment, peri-implant soft-tissue stability, and prosthetic survival, alongside patient-reported outcomes such as masticatory comfort and oral-health-related quality of life [89]. Third, subgroup analyses are needed to determine whether the laser-assisted advantage observed here in D2/D3 mandibular bone scales differently across other bone densities (D1 and D4), implant macrogeometries, anatomical sites (anterior vs. posterior mandible, maxilla), and timing scenarios (delayed vs. immediate placement). A formal cost-effectiveness analysis, parameterized with the survival and rehabilitation-time data from such follow-up studies, would then translate the early-stability advantage into health-economic outcomes that inform reimbursement decisions. Pending these confirmatory studies, the protocol described here can already be considered a clinically meaningful refinement of standard rotary osteotomy in carefully selected mandibular sites of D2/D3 bone density. In the mandibular arch specifically, biomechanical factors such as mandibular flexure under functional loading may further modulate peri-implant stress distribution and long-term implant stability in full-arch rehabilitations [90]; the contribution of such biomechanical variables to the stability advantage observed here warrants investigation in dedicated follow-up studies.

## 5. Conclusions

Within the limitations of this randomized controlled clinical study, the following conclusions can be drawn. Combining conventional rotary osteotomy with Er:YAG laser (λ = 2940 nm) biomodification of the implant site appears to have a synergistic effect on early implant stability, producing consistently higher ISQ values than rotary preparation alone at all time points assessed. Removal of the smear layer by Er:YAG laser is associated with significantly higher primary stability (Independent samples *t*-test, *p* < 0.05 at all time points), and the decline in implant stability between days 10 and 30—the so-called stability dip—was substantially attenuated in the case group compared with the controls; in the mesiodistal direction, the time × group interaction was statistically significant (F = 14.461, *p* < 0.001, partial η^2^ = 0.341). By day 30, ISQ values in the case group approached or equalled conventional secondary stability thresholds, supporting the clinical possibility of earlier loading in carefully selected mandibular D2/D3 sites. The high secondary stability observed in the cases, together with the absence of implant loss and the consistent stability advantage across both measurement directions, is consistent with favourable early implant stability dynamics and provides a basis for further prospective, multicentre research aimed at reducing rehabilitation time in dental implantology.

## 6. Patents

A patent entitled “MODULAR COMPLEX FOR PREPARATION OF THE IMPLANTOLOGY BED FOR SPIRAL DENTAL IMPLANT”, registration No. 4368 U1 of the Patent Office of the Republic of Bulgaria, resulted from the work reported in this manuscript.

## Figures and Tables

**Table 1:** Inclusion and exclusion criteria applied to all participants.

#	Inclusion Criteria	Exclusion Criteria
1	Age ≥ 18 years	Insufficient bone volume or density
2	Good general health; no severe systemic conditions	Active malignancy
3	Previously extracted mandibular premolar(s) or molar(s) (≥6 months prior)	Osteoporosis or previous radiotherapy to the jaws
4	Sufficient bone volume (premolar area); implant diameter ≤ 4.2 mm, length ≤ 11.5 mm; no augmentation required	Bisphosphonate, immunosuppressive, anticoagulant, or antiplatelet therapy
5	D2 or D3 bone density (Misch classification) on CBCT	Titanium hypersensitivity; pregnancy
6	Written informed consent provided	Active inflammatory disease of the oral cavity; mental illness; heavy smoking; significant family burden of implant failure

CBCT = cone-beam computed tomography; Misch D2/D3 = bone density classifications.

**Table 2:** Er:YAG laser settings used in the case group (Subtasks 1 and 2).

Parameter	Bone Biomodification (Cases, Subtasks 1 and 2)	Soft-Tissue Cap Excision (Cases, Subtask 2 Only)
Device	LiteTouch (Light Instruments Ltd., Yokneam, Israel)	LiteTouch (Light Instruments Ltd., Yokneam, Israel)
Wavelength (λ)	2940 nm	2940 nm
Program	Granulation Tissue Ablation Non-contact	Soft-tissue cutting
Pulse energy	400 mJ	200 mJ
Pulse frequency	17 Hz	35 Hz
Output power	6.80 W	7.00 W
Pulse duration	Pulsed (factory program)	Pulsed (factory program)
Water spray level	6	5–6
Tip	AS 7631 (X) Side-Firing, 1.3 × 19 mm	Crystal tip, 0.4 × 17 mm
Working mode	Non-contact	Contact
Working distance	≈1–2 mm from bone surface	Direct contact
Application time	2–3 min per quadrant (vestibular/oral)	Until full cap excision
Cooling	External, integrated water spray	External, integrated water spray

**Table 3:** Demographic and bone-quality characteristics of the study cohort (*N* = 90).

Variable	Statistic	Overall (*N* = 90)	Cases (*n* = 45)	Controls (*n* = 45)	*p*-Value
Number of patients	N	90	45	45	—
Age, years	Mean ± SE	50.99 ± 1.353	51.20 ± 1.92	50.78 ± 1.93	0.874 a
	Range (min–max)	21–77	23–76	21–77	—
Sex	Male, *n* (%)	47 (52.22)	24 (53.33)	23 (51.11)	0.830 b
	Female, *n* (%)	43 (47.78)	21 (46.67)	22 (48.89)	—
Bone density (Misch)	D2, *n* (%)	52 (57.78)	27 (60.00)	25 (55.56)	0.657 b
	D3, *n* (%)	38 (42.22)	18 (40.00)	20 (44.44)	—

a Independent samples *t*-test; b Chi-square test. All *p*-values > 0.05, confirming successful randomisation. Patients were randomized 1:1 into the case group (*n* = 45; rotary osteotomy + Er:YAG biomodification) and the control group (*n* = 45; rotary osteotomy alone). The two groups were comparable in age, sex, and bone density. Within each group, 30 patients (66.67%) underwent the closed protocol (Subtask 1) and 15 (33.33%) the open protocol (Subtask 2).

**Table 4:** Implant stability (ISQ) in the vestibulo-oral direction—cases vs. controls.

Time Point	Group	N	Mean	SE	SD	Mode	Min	Max
Primary VO	Cases	45	75.04	0.636	4.269	72	66	84
	Controls	45	72.29	0.504	3.382	72	65	80
Secondary VO	Cases	45	78.20	0.543	3.641	78	70	85
	Controls	45	74.82	0.514	3.446	72	69	82
Day 10 VO	Cases	15	77.60	1.050	4.067	78 a	69	83
	Controls	15	71.67	1.013	3.922	72	66	80
Day 20 VO	Cases	15	75.73	1.416	5.483	76 a	63	83
	Controls	15	65.67	0.860	3.331	68	61	72
Day 30 VO	Cases	15	77.07	1.395	5.405	80	63	84
	Controls	15	69.93	1.030	3.990	64	64	76

a Multiple modes exist; the smallest is reported.

**Table 5:** Implant stability (ISQ) in the mesiodistal direction—cases vs. controls.

Time Point	Group	N	Mean	SE	SD	Mode	Min	Max
Primary MD	Cases	45	76.49	0.639	4.289	76	67	84
	Controls	45	72.89	0.341	2.289	73	68	77
Secondary MD	Cases	45	79.73	0.461	3.093	80	72	85
	Controls	45	75.60	0.530	3.557	74	70	83
Day 10 MD	Cases	15	78.53	0.915	3.543	78	71	84
	Controls	15	71.47	1.009	3.907	69	65	80
Day 20 MD	Cases	15	76.80	1.353	5.240	80	64	84
	Controls	15	66.33	0.929	3.599	64	62	73
Day 30 MD	Cases	15	77.87	1.407	5.449	80	64	84
	Controls	15	69.93	1.030	3.990	68	64	76

**Table 6:** Proportion of implants by ISQ category—cases vs. controls (%).

Time Point/Direction	Group	ISQ < 70 (%)	ISQ ≥ 70 (%)
Primary VO	Cases	13.30	86.70
	Controls	22.20	77.80
Primary MD	Cases	8.90	91.10
	Controls	15.60	84.40
Secondary VO	Cases	0.00	100.00
	Controls	4.40	95.60
Secondary MD	Cases	0.00	100.00
	Controls	0.00	100.00
Day 10 VO	Cases	6.70	93.30
	Controls	40.00	60.00
Day 10 MD	Cases	0.00	100.00
	Controls	40.00	60.00
Day 20 VO	Cases	13.30	86.70
	Controls	86.70	13.30
Day 20 MD	Cases	13.30	86.70
	Controls	86.70	13.30
Day 30 VO	Cases	13.30	86.70
	Controls	53.30	46.70
Day 30 MD	Cases	13.30	86.70
	Controls	26.70	73.30

## Data Availability

The original contributions presented in the study are included in the article, further inquiries can be directed to the corresponding author.

## References

[1] Linkow L.I., Dorfman J.D. (1991). Implantology in dentistry. A brief historical perspective. N. Y. State Dent. J..

[2] Adell R. (1985). Tissue integrated prostheses in clinical dentistry. Int. Dent. J..

[3] Brånemark P.I. (1985). Introduction to osseointegration. Tissue Integrated Prostheses: Osseointegration in Clinical Dentistry.

[4] Zarb G., Albrektsson T. (1991). Osseointegration: A requiem for the periodontal ligament. Int. J. Periodont. Restor. Dent..

[5] Albrektsson T., Brånemark P.-I., Hansson H.-A., Lindström J. (1981). Osseointegrated titanium implants: Requirements for ensuring a long-lasting, direct bone-to-implant anchorage in man. Acta Orthop. Scand..

[6] Misch C.E. (1988). Bone character: Second vital implant criterion. Dent. Today.

[7] Lekholm U., Zarb G.A., Brånemark P.I., Zarb G.A., Albrektsson T. (1985). Patient selection and preparation. Tissue Integrated Prostheses: Osseointegration in Clinical Dentistry.

[8] Osman R.B., Swain M.V. (2015). A Critical Review of Dental Implant Materials with an Emphasis on Titanium versus Zirconia. Materials.

[9] Saini M., Singh Y., Arora P., Arora V., Jain K. (2015). Implant biomaterials: A comprehensive review. World J. Clin. Cases.

[10] Niinomi M. (2003). Recent research and development in titanium alloys for biomedical applications and healthcare goods. Sci. Technol. Adv. Mater..

[11] Pandey C., Rokaya D., Bhattarai B.P. (2022). Contemporary Concepts in Osseointegration of Dental Implants: A Review. BioMed Res. Int..

[12] Gehrke S.A., Treichel T.L.E., Aramburú Júnior J., de Aza P.N., Prados-Frutos J.C. (2020). Effects of the technique and drill design used during the osteotomy on the thermal and histological stimulation. Sci. Rep..

[13] Gehrke S.A., Aramburu Junior J.S., Perez-Albacete Martinez C., Ramirez Fernandez M.P., Mate Sanchez de Val J.E., Calvo-Guirado J.L. (2018). The influence of drill length and irrigation system on heat production during osteotomy preparation for dental implants: An ex vivo study. Clin. Oral Implants Res..

[14] Eriksson A.R., Albrektsson T. (1984). Assessment of bone viability after heat trauma. Scand. J. Plast. Reconstr. Surg..

[15] Sasaki K.M., Aoki A., Ichinose S., Yoshino T., Yamada S., Ishikawa I. (2002). Scanning electron microscopy and Fourier transformed infrared spectroscopy analysis of bone removal using Er:YAG and CO_2_ lasers. J. Periodontol..

[16] Mishra S.K., Chowdhary R. (2014). Heat generated by dental implant drills during osteotomy—A review: Heat generated by dental implant drills. J. Indian Prosthodont. Soc..

[17] Eriksson R.A., Adell R. (1986). Temperatures during drilling for the placement of implants using the osseointegration technique. J. Oral Maxillofac. Surg..

[18] Strbac G.D., Giannis K., Unger E., Mittlböck M., Vasak C., Watzek G., Zechner W. (2015). Drilling- and withdrawing-related thermal changes during implant site osteotomies. Clin. Implant Dent. Relat. Res..

[19] Augustin G., Davila S., Mihoci K., Udiljak T., Vedrina D.S., Antabak A. (2008). Thermal osteonecrosis and bone drilling parameters revisited. Arch. Orthop. Trauma Surg..

[20] Schwarz F., Sahm N., Becker J. (2014). Combined surgical therapy of advanced peri-implantitis lesions with concomitant soft tissue volume augmentation. A case series. Clin. Oral Implants Res..

[21] Vercellotti T. (2004). Technological characteristics and clinical indications of piezoelectric bone surgery. Minerva Stomatol..

[22] Stubinger S. (2010). Advances in bone surgery: The Er:YAG laser in oral surgery and implant dentistry. Clin. Cosmet. Investig. Dent..

[23] Pavlikova G., Foltan R., Horka M., Hanzelka T., Borunska H., Sedy J. (2011). Piezosurgery in oral and maxillofacial surgery. Int. J. Oral Maxillofac. Surg..

[24] Stacchi C., Bassi F., Troiano G., Rapani A., Lombardi T., Jokstad A., Sennerby L., Schierano G. (2020). Piezoelectric bone surgery for implant site preparation compared with conventional drilling techniques: A systematic review, meta-analysis and trial sequential analysis. Int. J. Oral Implantol..

[25] Preti G., Martinasso G., Peirone B., Navone R., Manzella C., Muzio G., Russo C., Canuto R.A., Schierano G. (2007). Cytokines and growth factors involved in the osseointegration of oral titanium implants positioned using piezoelectric bone surgery vs conventional rotary instruments. J. Periodontol..

[26] Vercellotti T., Crovace A., Palermo A., Molfetta L. (2001). The piezoelectric osteotomy in orthopedics: Clinical and histological evaluations (pilot study in anaimals). Mediterr. J. Surg. Med..

[27] Robiony M., Polini F., Costa F., Politi M. (2007). Ultrasonic bone cutting for surgically assisted rapid maxillary expansion (SARME) under local anaesthesia. Int. J. Oral Maxillofac. Surg..

[28] Aoki A., Mizutani K., Schwarz F., Sculean A., Yukna R.A., Takasaki A.A., Romanos G.E., Taniguchi Y., Sasaki K.M., Zeredo J.L. (2015). Periodontal and peri-implant wound healing following laser therapy. Periodontology 2000.

[29] Schwarz F., Aoki A., Sculean A., Becker J. (2009). The impact of laser application on periodontal and peri-implant wound healing. Periodontology 2000.

[30] Schwarz F., Olivier W., Herten M., Sager M., Chaker A., Becker J. (2007). Influence of implant bed preparation using an Er:YAG laser on the osseointegration of titanium implants: A histomorphometrical study in dogs. J. Clin. Periodontol..

[31] Lewandrowski K.U., Lorente C., Schomacker K.T., Flotte T.J., Wilkes J.W., Deutsch T.F. (1996). Use of the Er:YAG laser for improved plating in maxillofacial surgery: Comparison of bone healing in laser and drill osteotomies. Lasers Surg. Med..

[32] Pourzarandian A., Watanabe H., Aoki A., Ichinose S., Sasaki K.M., Nitta H., Ishikawa I. (2004). Histological and TEM examination of early stages of bone healing after Er:YAG laser irradiation. Photomed. Laser Surg..

[33] Kesler G., Romanos G., Koren R. (2006). Use of Er:YAG laser to improve osseointegration of titanium alloy implants—A comparison of bone healing. Int. J. Oral Maxillofac. Implants.

[34] Seymen G., Turgut Z., Berk G., Bodur A. (2013). Implant Bed Preparation with an Er, Cr: YSGG Laser Using Stereolithographic Surgical Guide. J. Lasers Med. Sci..

[35] Stubinger S., Nuss K., Pongratz M., Price J., Sader R., Zeilhofer H.F., von Rechenberg B. (2010). Comparison of Er:YAG laser, piezoelectric, and drill osteotomy for dental implant site preparation: A biomechanical and histological analysis in sheep. Lasers Surg. Med..

[36] Kanazirski N., Vladova D., Neychev D., Raycheva R., Kanazirska P. (2023). Effect of Er:YAG Laser Exposure on the Amorphous Smear Layer in the Marginal Zone of the Osteotomy Site for Placement of Dental Screw Implants: A Histomorphological Study. J. Funct. Biomater..

[37] Kanazirski N., Neychev D., Raycheva R., Zahariev N. (2023). Laser biomodification of the bone bed surface for placement of spiral dental implants: A study based on scanning electron microscopy. Folia Med..

[38] Meredith N. (1998). Assessment of implant stability as a prognostic determinant. Int. J. Prosthodont..

[39] Hao C.P., Cao N.J., Zhu Y.H., Wang W. (2021). The osseointegration and stability of dental implants with different surface treatments in animal models: A network meta-analysis. Sci. Rep..

[40] Swami V., Vijayaraghavan V., Swami V. (2016). Current trends to measure implant stability. J. Indian Prosthodont. Soc..

[41] Vollmer A., Saravi B., Lang G., Adolphs N., Hazard D., Giers V., Stoll P. (2020). Factors Influencing Primary and Secondary Implant Stability—A Retrospective Cohort Study with 582 Implants in 272 Patients. Appl. Sci..

[42] Sachdeva A., Dhawan P., Sindwani S. (2016). Assessment of Implant Stability: Methods and Recent Advances. Br. J. Med. Med. Res..

[43] Balshi S.F., Allen F.D., Wolfinger G.J., Balshi T.J. (2005). A Resonance Frequency Analysis Assessment of Maxillary and Mandibular Immediately Loaded Implants. Int. J. Oral Maxillofac. Implants.

[44] Staedt H., Kämmerer P.W., Goetze E., Thiem D.G.E., Al-Nawas B., Heimes D. (2020). Implant primary stability depending on protocol and insertion mode—An ex vivo study. Int. J. Implant Dent..

[45] Rabel A., Kohler S.G., Schmidt-Westhausen A.M. (2007). Clinical study on the primary stability of two dental implant systems with resonance frequency analysis. Clin. Oral Investig..

[46] Yeo I.S. (2014). Reality of dental implant surface modification: A short literature review. Open Biomed. Eng. J..

[47] Al-Sabbagh M., Eldomiaty W., Khabbaz Y. (2019). Can Osseointegration Be Achieved Without Primary Stability?. Dent. Clin. N. Am..

[48] Jemat A., Ghazali M.J., Razali M., Otsuka Y. (2015). Surface Modifications and Their Effects on Titanium Dental Implants. BioMed Res. Int..

[49] Yeo I.L. (2019). Modifications of Dental Implant Surfaces at the Micro- and Nano-Level for Enhanced Osseointegration. Materials.

[50] Sul Y.T., Johansson C.B., Röser K., Albrektsson T. (2002). Qualitative and quantitative observations of bone tissue reactions to anodised implants. Biomaterials.

[51] de Barros R.R., Novaes A.B., Korn P., Queiroz A., de Almeida A.L., Hintze V., Scharnweber D., Bierbaum S., Stadlinger B. (2015). Bone Formation in a Local Defect around Dental Implants Coated with Extracellular Matrix Components. Clin. Implant Dent. Relat. Res..

[52] Terheyden H., Lang N.P., Bierbaum S., Stadlinger B. (2012). Osseointegration—Communication of cells. Clin. Oral Implants Res..

[53] Gupta G. (2022). Implant Stability Quotient (ISQ): A Reliable Guide for Implant Treatment. Current Concepts in Dental Implantology—From Science to Clinical Research.

[54] Kasemo B., Lausmaa J. (1986). Surface science aspects on inorganic biomaterials. CRC Crit. Rev. Biocompat..

[55] Cohen J. (1988). Statistical Power Analysis for the Behavioral Sciences.

[56] Lakens D. (2013). Calculating and reporting effect sizes to facilitate cumulative science: A practical primer for *t*-tests and ANOVAs. Front. Psychol..

[57] Faul F., Erdfelder E., Lang A.-G., Buchner A. (2007). G*Power 3: A flexible statistical power analysis program for the social, behavioral, and biomedical sciences. Behav. Res. Methods.

[58] Hopewell S., Hirst A., Collins G.S., Altman D.G., Moher D., Schulz K.F., Tunn R., Aggarwal R., Berkwits M., Berlin J.A. (2025). CONSORT 2025 Statement: Updated guideline for reporting parallel group randomised trials. BMJ.

[59] Joos G. (1987). Theoretical Physics.

[60] Barfeie A., Wilson J., Rees J. (2015). Implant surface characteristics and their effect on osseointegration. Br. Dent. J..

[61] Peev S., Ever-Penov N. (2023). Dental Implantology.

[62] Berglundh T., Abrahamsson I., Lang N.P., Lindhe J. (2003). De novo alveolar bone formation adjacent to endosseous implants. Clin. Oral Implants Res..

[63] Ivanova V., Chenchev I., Zlatev S., Mijiritsky E. (2021). Correlation between Primary, Secondary Stability, Bone Density, Percentage of Vital Bone Formation and Implant Size. Int. J. Environ. Res. Public Health.

[64] Leucht P., Kim J.B., Wazen R., Currey J.A., Nanci A., Brunski J.B., Helms J.A. (2007). Effect of mechanical stimuli on skeletal regeneration around implants. Bone.

[65] Jimbo R., Tovar N., Anchieta R.B., Machado L.S., Marin C., Teixeira H.S., Coelho P.G. (2014). The combined effects of undersized drilling and implant macrogeometry on bone healing around dental implants: An experimental study. Int. J. Oral Maxillofac. Surg..

[66] Irandoust S., Müftü S. (2020). The interplay between bone healing and remodeling around dental implants. Sci. Rep..

[67] Perio D.N. (2013). Periodontal Bone Regeneration and the Er,Cr:YSGG Laser: A Case Report. Open Dent. J..

[68] Romanos G.E., Gutknecht N., Dieter S., Schwarz F., Crespi R., Sculean A. (2009). Laser wavelengths and oral implantology. Lasers Med. Sci..

[69] O’Donnell R.J., Deutsch T.F., Flotte R.J., Lorente C.A., Tomford W.W., Mankin H.J., Schomacker K.T. (1996). Effect of Er:YAG laser holes on osteoinduction in demineralized rat calvarial allografts. J. Orthop. Res..

[70] Stadlinger B., Ferguson S.J., Eckelt U., Mai R., Lode A.T., Loukota R., Schlottig F. (2012). Biomechanical evaluation of a titanium implant surface conditioned by a hydroxide ion solution. Br. J. Oral Maxillofac. Surg..

[71] Tettamanti L., Andrisani C., Bassi M.A., Vinci R., Silvestre-Rangil J., Tagliabue A. (2017). Immediate loading implants: Review of the critical aspects. Oral Implantol..

[72] Mello C.C., Lemos C.A., Verri F.R., dos Santos D.M., Goiato M.C., Pellizzer E.P. (2017). Immediate implant placement into fresh extraction sockets versus delayed implants into healed sockets: A systematic review and meta-analysis. Int. J. Oral Maxillofac. Surg..

[73] Sierra-Rebolledo A., Allais-Leon M., Maurette-O’Brien P., Gay-Escoda C. (2016). Primary Apical Stability of Tapered Implants Through Reduction of Final Drilling Dimensions in Different Bone Density Models: A Biomechanical Study. Implant Dent..

[74] Chou H.Y., Müftü S. (2013). Simulation of peri-implant bone healing due to immediate loading in dental implant treatments. J. Biomech..

[75] Stanford C.M. (2008). Surface modifications of dental implants. Aust. Dent. J..

[76] Kligman S., Ren Z., Chung C.H., Perillo M.A., Chang Y.C., Koo H., Zheng Z., Li C. (2021). The Impact of Dental Implant Surface Modifications on Osseointegration and Biofilm Formation. J. Clin. Med..

[77] Stadelmann W.K., Digenis A.G., Tobin G.R. (1998). Physiology and healing dynamics of chronic cutaneous wounds. Am. J. Surg..

[78] Fage S.W., Muris J., Jakobsen S.S., Thyssen J.P. (2016). Titanium: A review on exposure, release, penetration, allergy, epidemiology, and clinical reactivity. Contact Dermat..

[79] Klinger M.M., Rahemtulla F., Prince C.W., Lucas L.C., Lemons J.E. (1998). Proteoglycans at the bone-implant interface. Crit. Rev. Oral Biol. Med..

[80] Ponzetti M., Rucci N. (2021). Osteoblast Differentiation and Signaling: Established Concepts and Emerging Topics. Int. J. Mol. Sci..

[81] Brånemark P.I., Adell R., Albrektsson T., Lekholm U., Lundkvist S., Rockler B. (1983). Osseointegrated titanium fixtures in the treatment of edentulousness. Biomaterials.

[82] Kim J.M., Lin C., Stavre Z., Greenblatt M.B., Shim J.H. (2020). Osteoblast-Osteoclast Communication and Bone Homeostasis. Cells.

[83] Bornstein M.M., Scarfe W.C., Vaughn V.M., Jacobs R. (2014). Cone beam computed tomography in implant dentistry: A systematic review focusing on guidelines, indications, and radiation dose risks. Int. J. Oral Maxillofac. Implants.

[84] Trombelli L., Farina R., Tomasi C., Vignoletti F., Paolantoni G., Giordano F., Ortenai L., Simonelli A. (2024). Factors affecting radiographic marginal bone resorption at dental implants in function for at least 5 years: A multicenter retrospective study. Clin. Oral Implants Res..

[85] Chrcanovic B.R., Albrektsson T., Wennerberg A. (2014). Reasons for failures of oral implants. J. Oral Rehabil..

[86] Mangano F.G., Mangano C., Ricci M., Sammons R.L., Shibli J.A., Piattelli A. (2013). Esthetic evaluation of single-tooth Morse taper connection implants placed in fresh extraction sockets or healed sites. J. Oral Implantol..

[87] Mishra S., Chowdhary R. (2019). PEEK materials as an alternative to titanium in dental implants: A systematic review. Clin. Implant Dent. Relat. Res..

[88] Rahmitasari F., Ishida Y., Kurahashi K., Matsuda T., Watanabe M., Ichikawa T. (2017). PEEK with Reinforced Materials and Modifications for Dental Implant Applications. Dent. J..

[89] Wadhwani C.P.K., Schoenbaum T., King K.E., Chung K.H. (2018). Techniques to Optimize Color Esthetics, Bonding, and peri-implant Tissue Health with Titanium Implant Abutments. Compend. Contin. Educ. Dent..

[90] Caggiano M., D’Ambrosio F., Acerra A., Giudice D., Giordano F. (2023). Biomechanical Implications of Mandibular Flexion on Implant-Supported Full-Arch Rehabilitations: A Systematic Literature Review. J. Clin. Med..

